# Flexible, Low-Cost Sensor Based on Electrolyte Gated Carbon Nanotube Field Effect Transistor for Organo-Phosphate Detection

**DOI:** 10.3390/s17051147

**Published:** 2017-05-18

**Authors:** Vijay Deep Bhatt, Saumya Joshi, Markus Becherer, Paolo Lugli

**Affiliations:** 1 Department of Electrical Engineering and Information Technology, Institute for Nanoelectronics, Technische Universität München, Munich 80333, Germany; saumya.joshi@tum.de (S.J.); markus.becherer@tum.de (M.B.); 2 Faculty of Science and Technology, Free University of Bozen-Bolzano, 39100 Bolzano, Italy; Paolo.Lugli@unibz.it

**Keywords:** carbon nanotube, flexible, electrolyte gated transistor, organophosphate detection

## Abstract

A flexible enzymatic acetylcholinesterase biosensor based on an electrolyte-gated carbon nanotube field effect transistor is demonstrated. The enzyme immobilization is done on a planar gold gate electrode using 3-mercapto propionic acid as the linker molecule. The sensor showed good sensing capability as a sensor for the neurotransmitter acetylcholine, with a sensitivity of 5.7 μA/decade, and demonstrated excellent specificity when tested against interfering analytes present in the body. As the flexible sensor is supposed to suffer mechanical deformations, the endurance of the sensor was measured by putting it under extensive mechanical stress. The enzymatic activity was inhibited by more than 70% when the phosphate-buffered saline (PBS) buffer was spiked with 5 mg/mL malathion (an organophosphate) solution. The biosensor was successfully challenged with tap water and strawberry juice, demonstrating its usefulness as an analytical tool for organophosphate detection.

## 1. Introduction

Organophosphorus pesticides (OPs) are widely used in agriculture, and are potentially toxic for human health. Organophosphorus pesticides are esters, amides, or thiol derivatives of either phosphoric acid or thiophosphoric acid. The majority of pesticides now in use (e.g., malathion, azinphosmethyl, and chlorpyrifos) contain the thiono moiety (=S). The substitution of the =S for =O on the phosphorus atom increases the toxicity of the insecticide, such as in the case with malathion and its oxygen analogue, malaoxon [1].

Organophosphorus pesticides produce toxicity by inhibiting the enzyme acetylcholinesterase (AChE), which is responsible for the removal of the neurotransmitter acetylcholine (ACh) from the synaptic cleft through hydrolysis [2]. ACh acts as an excitatory transmitter for voluntary muscle in the somatic nervous system. OPs bind to AChE and produce a stable complex. In this process, the serine residue of AChE is blocked, causing a drastic accumulation of ACh at the nerve synapse, hence interfering with the normal nervous system function [3]. This results in rapid twitching of voluntary muscles, followed by paralysis. Designing a basic, rapid, and cheap analysis method for the detection of organophosphorus pesticides is of great importance. The traditional techniques for the detection of OPs are based on analytical methods such as spectrophotometry [4], spectrofluorimetry [5], infrared spectroscopy [6], photothermal [7], chemiluminescence [8], quartz crystal microbalance [9], mass spectrometry [10], and gas and/or liquid chromatography. Despite their high sensitivity, these methods have some disadvantages, such as long analysis times, as well as the need for expensive experimental instrumentation, trained personnel, and complex sample pretreatment. However, indirect bio-sensors can be used for monitoring OPs based on the modulated bio-catalytic activity of AChE [11,12,13,14,15]. After the exposure of the enzyme AChE to the OP, its catalytic/enzyme activity is inhibited, and this decreased enzyme activity can permit a quantification of OP; thus, such sensors get the name *inhibition sensors* [11].

In 1970, the first ion-sensitive field effect transistor (ISFET)-based pH sensor was developed by P. Bergveld [16]. Later, in 1976, Caras and Janata integrated enzymes into ISFET for the detection of penicillin [17]. Since then, tremendous progress has been made in the area of field effect transistor (FET)-based enzymatic sensors. These enzymatic biosensors have aroused great interest among the scientific community due to their excellent sensitivity, selectivity towards analytes, easy measurement procedure, quick response, and potential for miniaturization [18,19].

Inherent properties of carbon nanotubes (CNTs) like high surface area, acceptable biocompatibility, chemical and electrochemical stability, and good electrical conductivity make them suitable candidates for chemical/bio-sensing applications. There are reports [20,21] where carbon nanotube electrodes are used for the detection of organophosphate pesticides. As the enzyme acetylcholineesterase also hydrolyzes the neurotransmitter acetylcholine, the same biosensor can be used for acetylcholine detection. Dysfunctional ACh regulation in the brain causes several disorders, such as Parkinson’s disease, Alzheimer’s disease, and myasthenia gravis [22]; therefore, measuring the concentration of acetylcholine in the brain fluid has a great clinical importance. There are also reports where CNT-based electrodes [21,23] are used for the detection of acetylcholine. Researchers have also tried to use carbon nanotube field effect transistors (CNTFETs) for the detection of acetylcholine by modifying active semiconducting CNTs; for example, in receptor-modified CNTs [24], self-assembly of CNTs with nanoparticles [25], or self-assembly of CNTs and AChE [22]. There is also a report of a dual-gated FET configuration [26] for the detection of acetylcholine. In this paper, we report a flexible spray-deposited CNTFET-based enzymatic sensor for the detection of organophosphorus pesticide. The sensor is demonstrated to be a potential acetylcholine sensor, and can resolve concentrations as low as in the pM range. The selectivity tests of the sensor are performed against serine and lysine neurotransmitters. We also performed the mechanical bending tests for the flexible sensor to verify its capability to withstand mechanical deformations, owing to its potential use as an implantable device for neurotransmitter detection or as a pesticide detector in the food packaging industry.

Malathion (an organophosphate pesticide) is widely used in crops such as strawberries, cranberries, apples, peaches, etc. to protect them from insects like aphids, spittlebugs, and plantbugs [27]. Malathion degrades very rapidly in air, with a half-life of approximately 5 h. In water, the degradation rate is highly dependent on the pH and organic content. At higher pH (>6–7) the complete degradation of malathion may occur in 6–7 days; however, if the pH is low (<5) and the organic content is little, malathion may persist with a half-life of months or even years [28]. Thus, malathion is used to study the inhibition percentage of the enzyme acetylcholinesterase with time. For real sample analysis, tap water and freshly-squeezed strawberry juice are spiked with a known amount of malathion.

## 2. Materials and Methods

### 2.1. Materials

The chemicals used for device functionalization were: acetylcholinesterase (AChE) from *Electrophorus electricus* (electric eel) Type VI-S (EC 3.1.1.7), N-(3-dimethylaminopropyl)-N’ ethylcarbodiimide hydrochloride (EDC), N-hydroxy-sulfo-succinimide (sulfo-NHS), and 3-mercaptopropionic acid (99%). Semiconducting CNTs (90%) of mean diameter 0.77 nm and average length 770 nm were used as active materials. Buffer solutions were prepared with phosphate-buffered saline (PBS) pH = 7.4. Acetylcholine chloride (99%) solutions in PBS buffer were used to test the sensor. The organophosphate malathion (Pestanal^®^) was used for the inhibition study. All the chemicals were purchased from Sigma-Aldrich (Munich, Germany).

### 2.2. Fabrication of CNTFET

**Figure 1:**
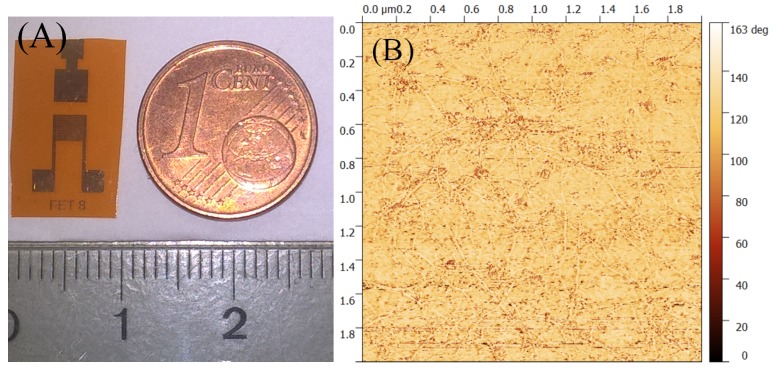
(**A**) Image of the planar carbon nanotube field effect transistor (CNTFET). (**B**) Atomic force microscopy (AFM) image (phase image) of the random CNT network.

The image of the planar CNTFET is shown in Figure 1A. To achieve patterns of source, drain, and gate contacts, standard negative photolithography followed by liftoff process was performed. The channel width/length or the aspect ratio was 900 and the channel length was 50 μm for the CNTFETs. Thermally-evaporated 5 nm-thick Cr was used as an adhesion promoter layer, followed by a 40 nm-thick Au layer to form the source, drain, and gate contacts. Aqueous medium surfactant sodium dodecyl sulfate (SDS) was used to disperse the semiconducting CNTs, and this solution was sprayed using a shadow mask to form the active channel [29]. The automated spray system used for spraying was equipped with an industrial air atomizing spray valve (Nordson EFD, East Providence, RI, USA) in combination with an overhead motion platform (Precision Valve & Automation, Cohoes, NY, USA). More details about the spray deposition process set-up can be found elsewhere [30,31]. The samples were immersed for 15 min in DI-H${}_{2}$O to remove surfactant from the single-walled CNT (SWCNT) network. Figure 1B shows the atomic force microscopy (AFM) image of the CNT channel. This is the phase AFM image taken in non-contact mode. As seen in the AFM image, the CNT network was random in nature, and therefore the percolation path of the current was probabilistic in nature. This results in variations in the transistor characteristics from one device to another.

### 2.3. Enzyme Immobilization Scheme

**Figure 2:**
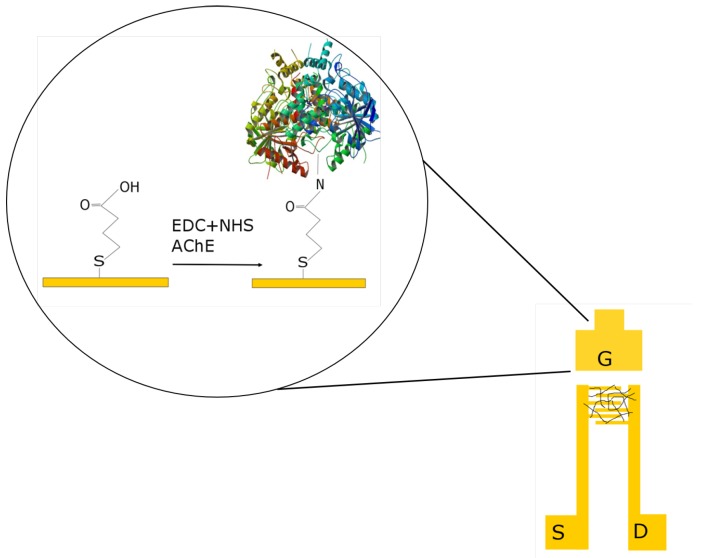
Schematic of the functionalization scheme. The gold gate is functionalized with acetylcholinesterase using 3-MPA as a linking molecule.

Because of their high affinity to gold, alkanethiols are widely used to fabricate modified self-assembled monolayer (SAM) electrodes in amperometric sensors [32]. These well-ordered monolayers of alkanethiols can then be used to immobilize an enzyme. Amperometric biosensors where different enzymes are immobilized by using 3-mercaptopropionic acid (MPA) self-assembled monolayer have been proposed in the literature [33,34]. In this work, we use the MPA linker molecule to immobilize the enzyme AchE onto the planar gold contact surface of the CNTFET. Figure 2 describes the enzyme immobilization scheme.

On the planar gate surface, 25:75 (*v/v*) water/ethanol solution containing 70 mM MPA was placed for approximately 9 h to form the Au-MPA SAM. Samples were then washed thoroughly with the same 1:3 ethanolic solution and air dried. EDC/NHS activation chemistry was used to activate the carboxylic groups, and 30 μL each of 30 mM EDC and 30 mM NHS was locally dropped on the gold gate surface and left for four hours. The samples were again thoroughly washed with PBS buffer, and immediately 50 μL of 2 mg/mL AChE was dropped on the activated gate area. The samples were kept for drying at room temperature for around 12 h or until the liquid evaporated. Finally, the samples were washed with PBS buffer and stored at 4 °C when not in use.

**Figure 3:**
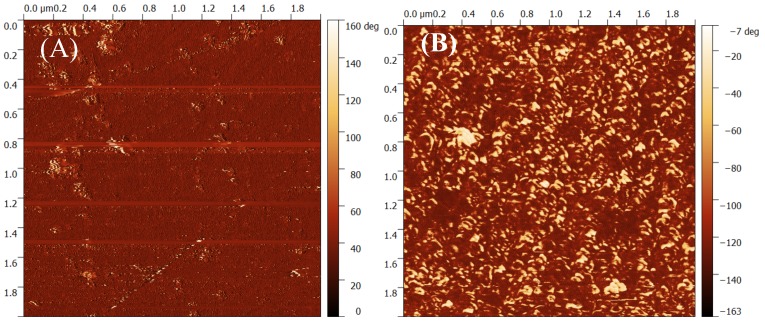
Phase AFM image of the gold gate (**A**) before, and (**B**) after the immobilization of enzyme acetylcholinesterase (AChE). D: drain; G: gate; S: source; EDC: N-(3-dimethylaminopropyl)-N’ ethylcarbodiimide hydrochloride; NHS: N-hydroxysuccinimide.

Figure 3 shows the AFM phase image of the gold gate contact before and after the enzyme AChE is immobilized on the surface.

## 3. Results

**Figure 4:**
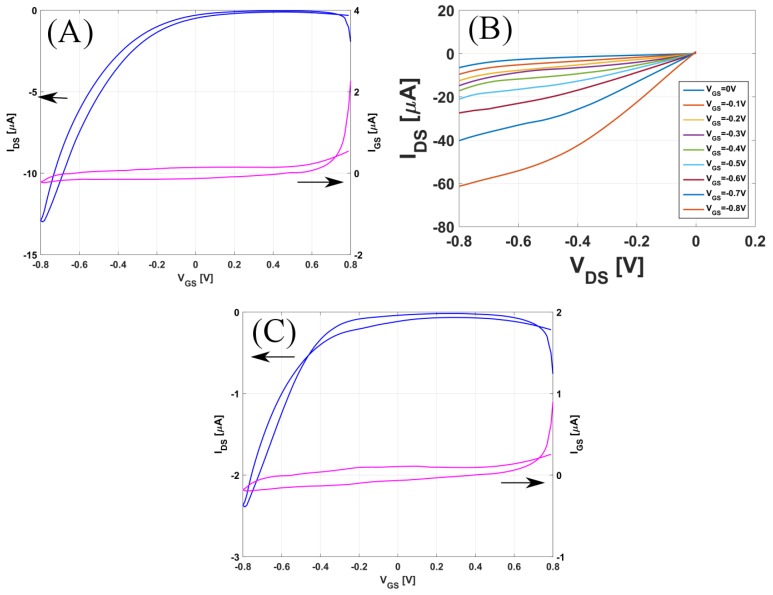
(**A**) Transfer curve and (**B**) Output Curve for the un-functionalized CNTFET; (**C**) Transfer curve for the functionalized CNTFET.

The sensors were electrically characterized using a Keithley Semiconductor Parameter Analayzer 4200 (Tektronik, Munich, Germany). For all electrical measurements, a Polydimethylsiloxane(PDMS) chamber was mounted around the active area of the CNTFETs to serve as a compartment for 60 μL of the analyte solution, which was exchanged manually using a Gilson pipette. Figure 4 shows transistor characteristics (transfer and output curves) for the CNTFET, recorded in 1 mM PBS buffer solution. Figure 4A records both the forward and backward sweep of the transfer curve for the un-functionalized CNTFET; the gate-to-source voltage (V${}_{GS}$) was swept from 0.8 V to −0.8 V, and the drain-to-source voltage (V${}_{DS}$) was fixed to −0.1 V. The device had a typical p-type behavior with an on–off ratio of around 350. Figure 4B records the output curves, where drain-to-source voltage was swept from 0 to −0.8 V with 10 mV step for different gate-to-source voltages varying from 0 to −0.8 V. Figure 4C shows the transfer curve for the functionalized CNTFET. The on/off ratio after functionalization was around 150, which is half of the unfunctionalized transistor.

### 3.1. Acetylcholine Sensor

**Figure 5:**
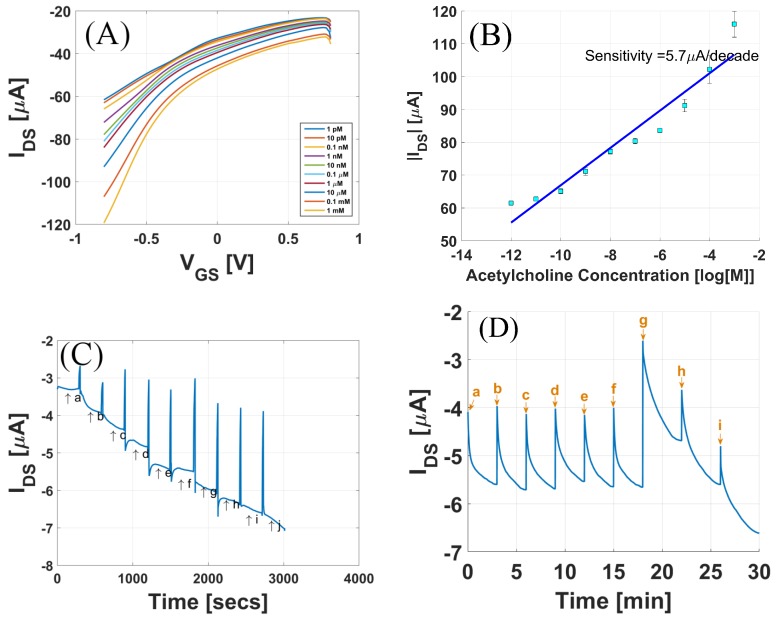
(**A**) Response of the sensor to different acetylcholine concentration; (**B**) Drain Current (I${}_{DS}$) vs. acetylcholine concentration; (**C**) Real-time response of the sensor from 1 pM to 1 mM concentration range. a = 1 pM, b = 10 pM, c = 0.1 nM, d = 1 nM, e = 10 nM, f = 0.1 μM, g = 1 μM, h = 10 μM, i = 0.1 mM, j = 1 mM. (**D**) Selectivity tests for sensor in the presence of serine and glycine.

The sensor is first demonstrated as a neurotransmitter acetylcholine sensor. Figure 5A shows the response of the sensor to different concentrations of acetylcholine. For these measurements, (V${}_{DS}$) was fixed to −0.2 V, and (V${}_{GS}$) was swept from +0.8 V to −0.8 V. The measurements were started from 1 pM acetylcholine, and three runs of transfer curves were recorded, keeping the concentration constant. Figure 5A shows the average of the three runs. Figure 5B shows the drain-to-source current as a function of the concentration of acetylcholine for fixed applied biases (V${}_{DS}$) = −0.2 V and (V${}_{GS}$) = −0.8 V. The effect of increasing acetylcholine concentration on the gate current was negligible, as shown in Figure S1. To calculate the sensitivity of the sensor, a linear fit of the curve was performed using the least square fit. The slope was extracted, yielding the sensitivity of the sensor, which in this case is 5.7 μA/decade. Real-time response of the sensor for the fixed applied biases (V${}_{DS}$) = −0.2 V and (V${}_{GS}$) = −0.8 V in the concentration range from 1 pM to 1 mM is shown in Figure 5C. We observe a continuous increase in the drain-to-source current as the concentration of acetylcholine is increased.

As there are many interfering analytes present in body fluids, it is important that the sensor is selective to only acetylcholine changes. To examine the selectivity of the biosensor, different solutions were prepared containing variations in the concentration of acetylcholine, serine, and glycine. The concentrations of the analytes were chosen to approximate their physiological concentrations. The composition of different solutions labelled a–i mentioned in Figure 5D are given in Table 1. Solutions a–c and d–f had varying concentrations of serine and glycine, respectively, while acetylcholine concentration was fixed. Solutions g, h, and i had varying concentrations of acetylcholine, while serine and glycine concentrations were fixed. From Figure 5D it is clear that the sensor responded to changes in the concentration of acetylcholine, with almost no response to varying concentrations of interfering analytes.

**Figure 6:**
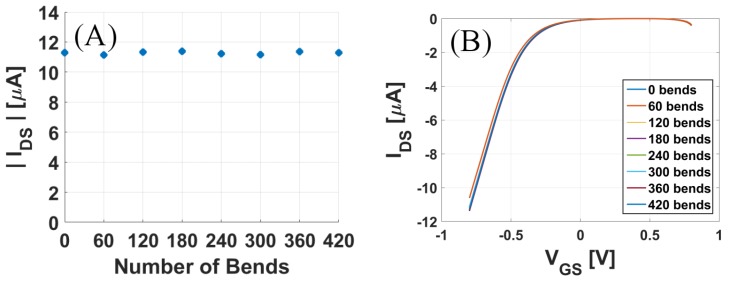
Flexibility tests for the acetylcholine sensor: (**A**) Maximum drain current and (**B**) Transfer curve, recorded for 10 nM acetylcholine after every 60 bending cycles.

One of the primary advantages of flexible sensors is that they can withstand mechanical deformation. To test their mechanical endurance, the sensors were flexed to a 90° angle at the radius of 0.17 cm with a speed of 50 mm/s for more than 400 total iterations. After every 60 iterations, the response of the sensor to 10 nM acetylcholine solution was recorded. As seen in Figure 6A,B, the current response was well maintained after such repeated bending cycles, indicating the robustness of the sensors against mechanical deformation or stress.

### 3.2. Inhibition of Acetylcholinesterase Activity with the Organophosphate Malathion

**Figure 7:**
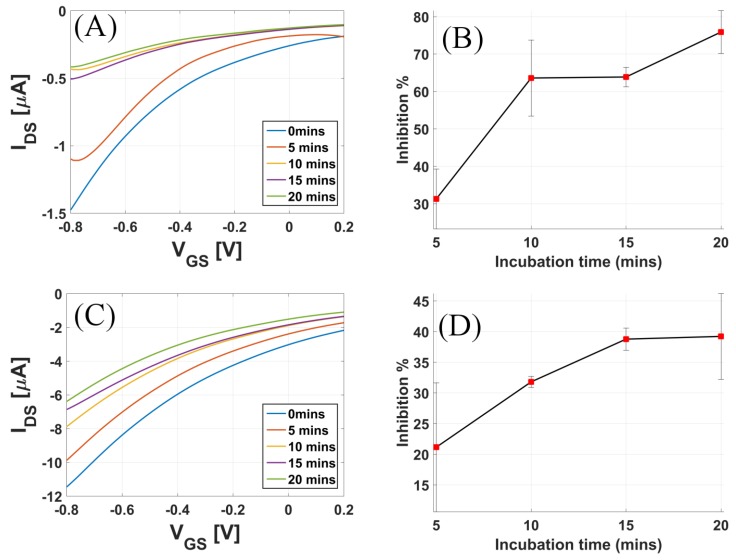
Transfer curves for increasing incubation time when the sensor was exposed to (**A**) 5 mg malathion and (**C**) 2 mg malathion solutions in phosphate-buffered saline (PBS). Inhibition % of enzyme activity when the sensor was exposed (**B**) 5 mg malathion and (**D**) 2 mg malathion solutions in PBS.

Solutions of the organophosphate Malathion in 1 mM PBS buffer solutions were prepared. The inhibition study was performed by incubation method. The drain-to-source current (I${}_{DS}$) of the biosensor was measured for 1 nM acetylcholine concentration before it was exposed to malathion solution. The applied biases were V${}_{DS}$ = −0.1 V and V${}_{GS}$ = −0.8 V, and this I${}_{DS}$ is the called I${}_{DS,Control}$. The sensor was then exposed to malathion from 5 to 20 min (incubation time) at an interval of 5 min. Figure 7A shows the transfer curve for the different incubation times, and Figure 7B shows the inhibition of the enzyme activity (Inhibition %), calculated as follows:

(1) $$Inhibition\%=\frac{I_{DS,Control}-I_{DS}}{I_{DS,Control}}\times 100$$

Here I${}_{DS}$ is the drain-to-source current measured in 1 nM acetylcholine solution after the sensor was exposed to 5 mg/mL malathion solution in PBS buffer for the stated amount of time. To investigate the impact of varying the concentration of malathion, an inhibition study was again performed by exposing the sensors to 2 mg/mL malathion solution. Figure 7C shows the transfer curves for increasing incubation times, and Figure 7D shows the inhibition % vs. incubation time when the sensors were exposed to 2 mg malathion solution.

### 3.3. Real Sample Analysis

**Figure 8:**
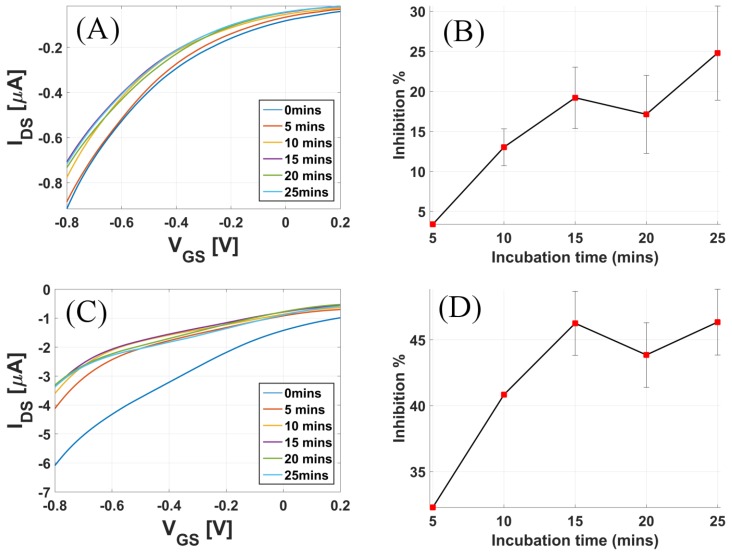
Real sample analysis: Transfer curves for increasing incubation time when the sensor was exposed to (**A**) 1.35 mg malathion in 1 mL tap water and (**C**) 1.35 mg malathion mixed in 1 mL strawberry pulp. Inhibition% of enzyme activity when the sensor was exposed to (**B**) 1.35 mg malathion in 1 mL tap water and (**D**) 1.35 mg malathion mixed in 1 mL strawberry pulp.

To analyze the efficiency of these sensors in real use, samples of tap water and strawberry juice were used. Malathion is one of the common pesticides used in strawberry crops [27]. Fresh strawberries were bought from a local supermarket and fresh juice was extracted, which was then spiked with malathion solutions to obtain 1.35 mg/mL concentration. Similarly, tap water was spiked with solutions of malathion to obtain 1.35 mg/mL concentration. No prior filtration step was performed. The incubation method as explained above was used for the inhibition study in real sample analysis. Figure 8A shows the plot of the inhibition % vs. incubation time for tap water. The maximum inhibition % for tap water was below 25%, reached in 15 min, and 45% for strawberry samples, reached in 20 min.

## 4. Discussion

Figure 5 summarizes the results of the biosensor used for sensing the neurotransmitter acetylcholine. Figure 5A shows that as the concentration of acetylcholine increased from 1 pM to 1 mM, there was a continuous increase in the drain-to-source current. AChE hydrolyses ACh into choline and acetic acid, releasing H${}^{+}$ ions into the electrolyte solution, in the presence of H${}_{2}$O. The hydrolysis of ACh can be summarized in the equation below:

(2) $$\begin{array}{c} CH_{3}COO{\left(CH_{2}\right)}_{2}N^{+}{\left(CH_{3}\right)}_{3}+H_{2}O\overset{AChE}{\to }{\left(CH_{3}\right)}_{3}{\left(CH_{2}\right)}_{2}NOH+CH_{3}COOH+H^{+} \end{array}$$

(3) $$\begin{array}{c} CH_{3}COOH\overset{\;}{⇌}CH_{3}COO^{-}+H^{+} \end{array}$$

As ACh solution is added, the AChE immobilized on the gold gate catalyzes the above reaction. The H+ ions released affect the surface potential of the gate surface. As the positive charge near the gate surface increases, this induces an increased negative charge near the CNT channel, hence increasing the p-doping of the CNT channel. An increased doping gives rise to an increase in the drain-to-source current for a p-type device. For reference, the pH dependence of a CNTFET is also shown in Figure S2. As seen in Figure 5, the sensor is highly sensitive in the lower concentration range and can resolve concentrations as low as 1 pM. To calculate the sensitivity, I${}_{DS}$ vs. concentration of ACh was plotted for fixed applied biases. The sensor had an almost linear profile in the concentration range from 1 pM to 1 mM. Using the linear fitting model in Matlab, the calculated sensitivity was 5.6855 μA/decade for this device. Incubation time is the reaction time of the enzyme with the inhibitor [35]. The results from Figure 7 show that the degree of enzyme inhibition usually increases with the incubation time, until reaching a plateau [36]. The highest degree of inhibition was not 100%, which is most likely attributed to the binding equilibrium between pesticide and binding sites in enzyme [37]. The saturation of enzyme activity is also dependent on the concentration of malathion used for exposure. As seen in Figure 7A, there was saturation at around 65% enzyme activity after the sensor was incubated for 10 min when exposed to 5 mg malathion. However, when the sensor was exposed to 2 mg malathion, the inhibition % saturated after a longer incubation time. It is also important to note from Figure 7 that the inhibition was maximal for the initial incubation times, and for longer incubation times, the inhibition rate was lower.

## 5. Conclusions

In this work, an AChE biosensor for the detection of organophosphorus insecticides based on enzyme inhibition method was demonstrated. The potential use of this biosensor as a sensor for the neurotransmitter acetylcholine is also shown. The enzyme was immobilized on the gold gate contact pad of a planar carbon nanotube field effect transistor using a linker molecule. The device was fabricated using a low-cost spray deposition technique on a flexible polyimide substrate. Several experiments were performed to demonstrate the high sensitivity and specificity of the sensors. As the final sensor is intended for food packaging, mechanical endurance tests were also performed. The biosensor was challenged for organophosphorus detection in PBS buffer spiked with malathion, and gave impressive results. For real sample analysis, the inhibition of enzyme activity was measured against malathion in strawberry juice and tap water.

## Figures and Tables

**Table 1:** Composition of solutions used for selectivity tests (refer to Figure 5D).

Individual Concentrations of Species (μM)			
Solution	Serine	Glycine	Acetylcholine
a	0.2	20	50
b	2	20	50
c	20	20	50
d	20	0.2	50
e	20	2	50
f	20	20	50
g	20	20	5
h	20	20	50
i	20	20	100

## Supplementary Materials

The following are available online at http://www.mdpi.com/1424-8220/17/5/1147/s1, Figure S1: Impact of increasing acetylcholine concentration on the gate current, Figure S2: pH dependence of CNTFETs, Figure S3: Control experiement1: Response of an un-functionalized device to varying concentrations of acetylcholine, Figure S4: Control experiment2: Response of an un-functionalized device to varying concentrations of acetic acid.
