# Supplementary material for: Flexible, Low-Cost Sensor Based on Electrolyte Gated Carbon Nanotube Field Effect Transistor for Organo-Phosphate Detection
Source: Sensors (Basel). 2017 May 18;17(5):1147. doi: 10.3390/s17051147 (PMC5470893; doi:10.3390/s17051147)
Supplement: Supplementary file 1 [file sensors-17-01147-s001.pdf]

Supplementary Materials: Flexible, Low-Cost Sensor Based on Electrolyte Gated Carbon Nanotube Field Effect Transistor for Organo-Phosphate Detection

Vijay Deep Bhatt, Saumya Joshi, Markus Becherer and Paolo Lugli

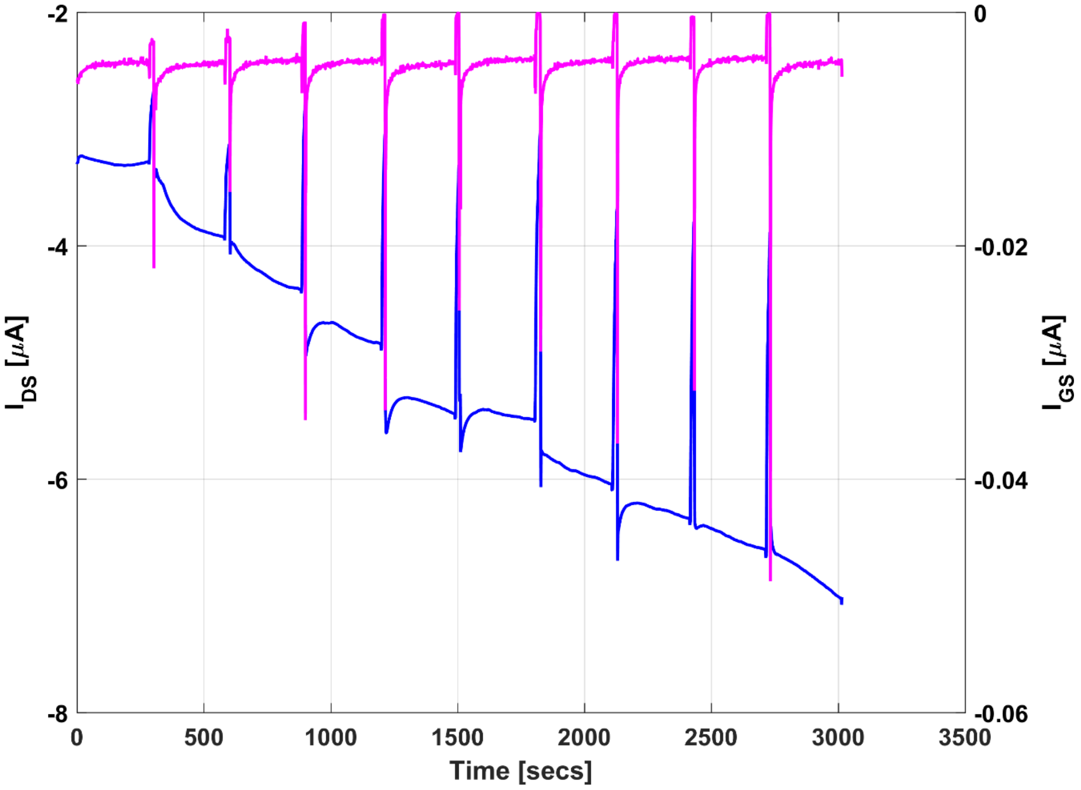

Figure S1. Impact of increasing acetylcholine concentration on the gate current.

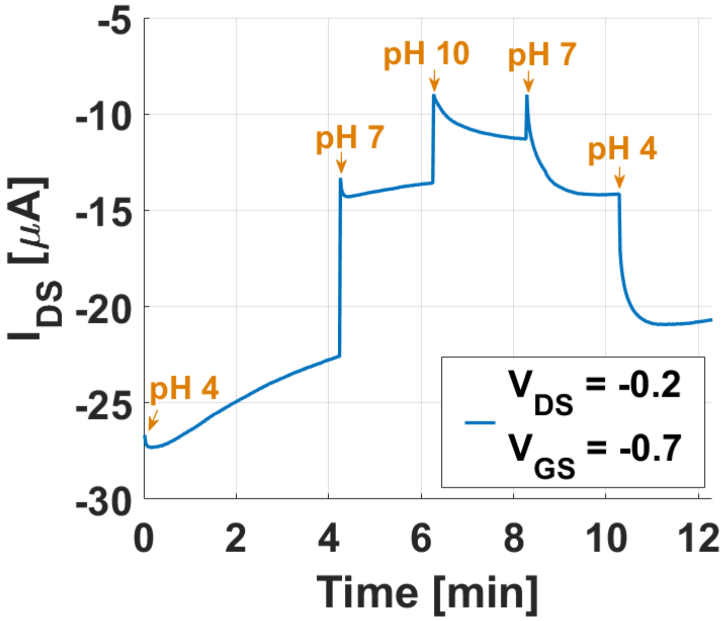

Figure S2. pH dependence of CNTFETs.

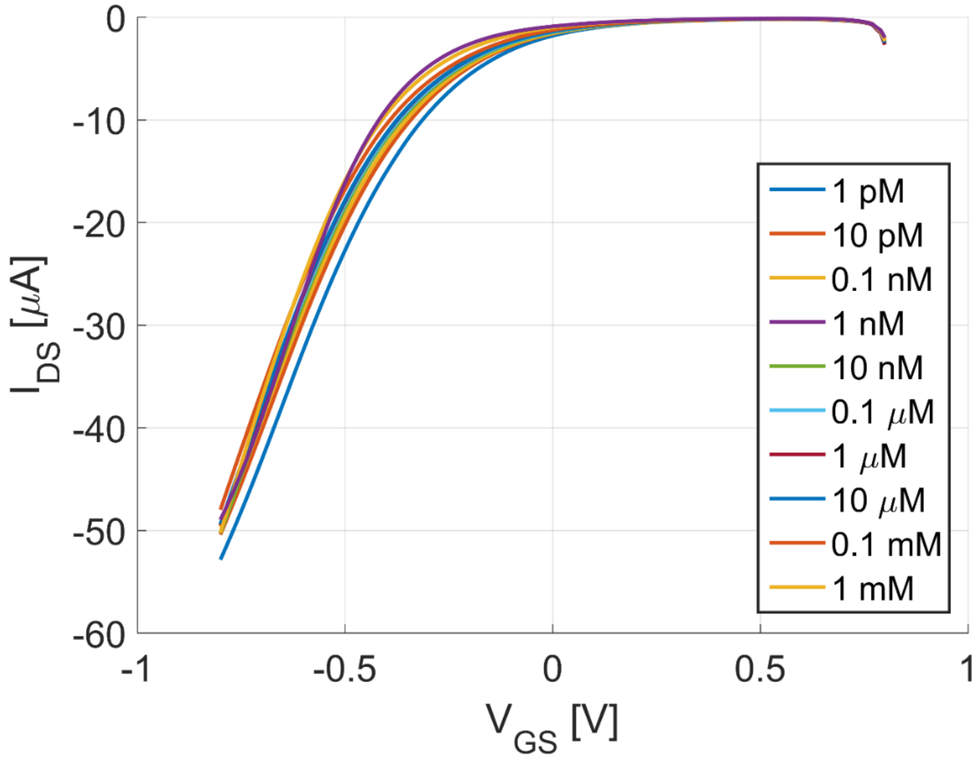

Figure S3. Control experiement1: Response of an un-functionalized device to varying concentrations of acetylcholine.

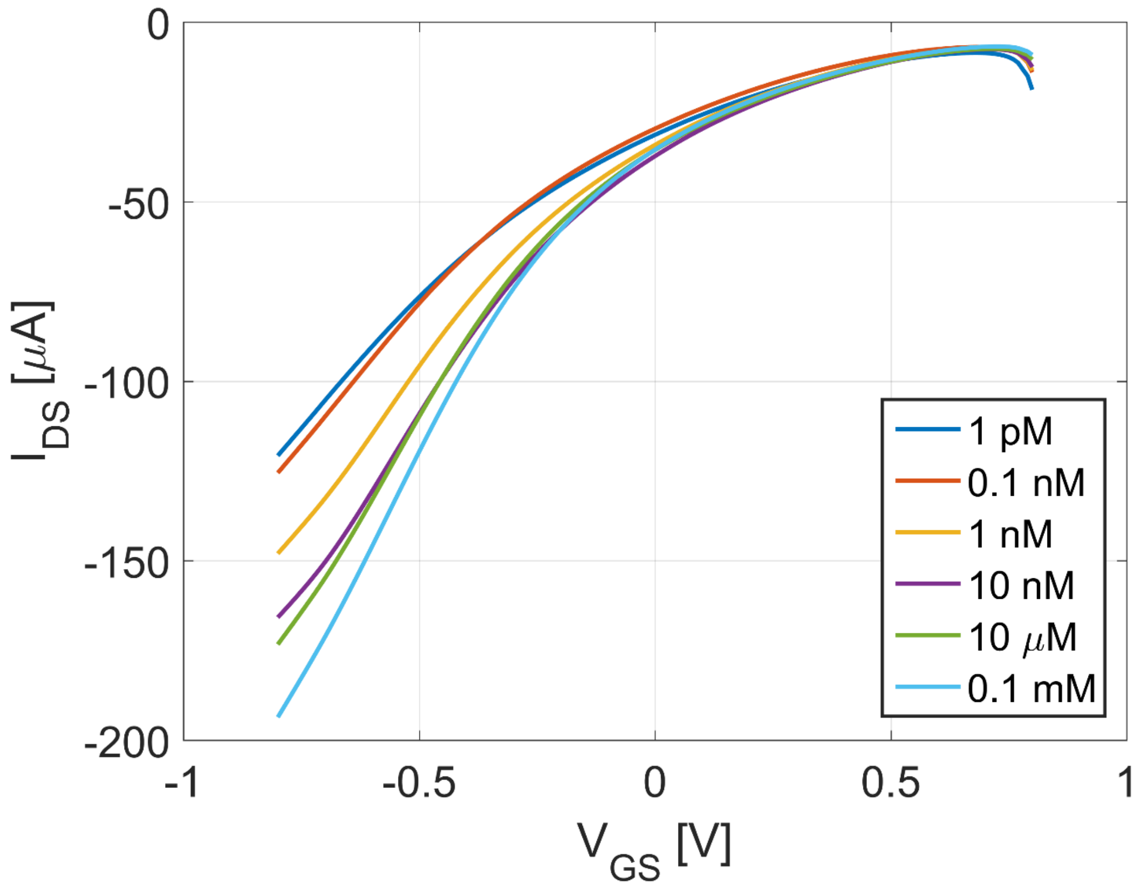

Figure S4. Control experiment2: Response of an un-functionalized device to varying concentrations of acetic acid.
